# Integrated olfactory receptor and microarray gene expression databases

**DOI:** 10.1186/1471-2105-8-231

**Published:** 2007-06-30

**Authors:** Nian Liu, Chiquito J Crasto, Minghong Ma

**Affiliations:** 1 Center for Medical Informatics, Yale University School of Medicine, New Haven, CT 06520, USA; 2 Department of Anesthesiology, Yale University School of Medicine, New Haven, CT 06520, USA; 3 Department of Neurobiology, Yale University School of Medicine, New Haven, CT 06520, USA; 4 Department of Neuroscience, University of Pennsylvania School of Medicine, Philadelphia, PA 19104, USA; 5 Department of Genetics, University of Alabama at Birmingham, Birmingham, AL 35294, USA

## Abstract

**Background:**

Gene expression patterns of olfactory receptors (ORs) are an important component of the signal encoding mechanism in the olfactory system since they determine the interactions between odorant ligands and sensory neurons. We have developed the Olfactory Receptor Microarray Database (ORMD) to house OR gene expression data. ORMD is integrated with the Olfactory Receptor Database (ORDB), which is a key repository of OR gene information. Both databases aim to aid experimental research related to olfaction.

**Description:**

ORMD is a Web-accessible database that provides a secure data repository for OR microarray experiments. It contains both publicly available and private data; accessing the latter requires authenticated login. The ORMD is designed to allow users to not only deposit gene expression data but also manage their projects/experiments. For example, contributors can choose whether to make their datasets public. For each experiment, users can download the raw data files and view and export the gene expression data. For each OR gene being probed in a microarray experiment, a hyperlink to that gene in ORDB provides access to genomic and proteomic information related to the corresponding olfactory receptor. Individual ORs archived in ORDB are also linked to ORMD, allowing users access to the related microarray gene expression data.

**Conclusion:**

ORMD serves as a data repository and project management system. It facilitates the study of microarray experiments of gene expression in the olfactory system. In conjunction with ORDB, ORMD integrates gene expression data with the genomic and functional data of ORs, and is thus a useful resource for both olfactory researchers and the public.

## Background

The mammalian olfactory system, with its ability to detect numerous odor molecules in the environment, helps animals locate food, mates, and predators. The detection sensitivity and specificity largely depend on the olfactory receptors (ORs) expressed in the primary sensory neurons within the neuroepithelium in the nose [1]. In rodents, there are approximately 10 million sensory neurons expressing > 1000 different types of OR genes in each nostril [2]. Each neuron expresses only one specific OR, which exhibits an optimal response to specific odorants [3]. Axons from the sensory neurons that express the same OR converge onto one or a few glomeruli per olfactory bulb [4]. The expression patterns of OR genes and the unique projection of their sensory neurons provide a molecular basis for olfactory signal coding in the brain.

Diverse sets of data have resulted from olfactory research carried out at the cellular, functional and behavioral levels [5-9]. Since the first identification of rat OR genes in 1991, thousands of ORs have been found, some in tissues not associated with the olfactory system [10,11]. Functional imaging in the olfactory bulb has produced two-dimensional "odor maps" (unique activity patterns) in the glomerular layer [12-14]. Three key databases in the SenseLab [ORDB (OR database), OdorDB (odor database) and OdorMapDB (odor map database)], housed at the Yale University School of Medicine, provide an integrated, multidisciplinary model for the olfactory pathway [15-18]. The information contained in these databases illustrates the chain of events that starts from the exposure of the animals to odorous environment, to the binding of odorant ligands with specific ORs, and to the resulting spatial activity patterns in the olfactory bulb.

Recent microarray studies using Affymetrix (Santa Clara, CA) gene-chips have resulted in the accumulation of a large amount of OR gene expression data. These data show the differential expression of OR genes in the olfactory and non-olfactory tissues [11]. Building an efficient microarray database specific for the ORs and integrating it with the SenseLab system has been challenging. There are many large-scale and well-established databases, e.g., ArrayExpress at the European Bioinformatics Institute [19], the Stanford Microarray Database [20], and the Gene Expression Omnibus (GEO) at the National Center for Biotechnology Information (NCBI) [21], for public data repositories of the microarray experiments. However, they do not fit the needs specific to the chemosensory research community, i.e., to have a domain-specific database integrated with the SenseLab system.

This paper describes the Olfactory Receptor Microarray Database (ORMD), an informatics tool dedicated to disseminating information related to rodent OR microarray experiments. Currently archived in the ORMD are gene expression data of ORs in the olfactory epithelium as well as other tissues. ORMD is integrated with ORDB (which relies on an architecture that is common to all databases in SenseLab), through dynamic link in the webpage for each OR. Both databases are designed to facilitate the experimental research on elucidating the mechanisms underlying perception of smell.

## Construction and content

### Methods

The database schema of ORMD has been implemented in Oracle. It is primarily a traditional relational database. Some tables, however, implement the EAV (Entity-Attribute-Value) data model [22]. Since the number and names of the parameters defining the experimental conditions may vary and be unpredictable, the EAV model has been chosen to store the values of some parameters for the experiments. This model ensures flexibility of the system. Also, an EAV-like approach has been used to store gene expression datasets, with the experiment being the entity, the probe set name being the attribute, and the signal, detection call, and P-value being the values. The Web application has been developed in Java Server Pages (JSP) and run on Apache Tomcat Server.

### Olfactory Receptor Database – ORDB

ORDB is one of the major databases in the SenseLab system [23]. As previously reported [18], the archived OR properties are categorized as descriptive attributes and sequence data. The descriptive attributes include animal species name (i.e., organism), strain, source tissue, chromosomal location, data source, sequence laboratories [the principal investigator's (PI's) lab that cloned the OR gene or identified the gene from the genome], and references (including links to GenBank and Medline sources for the OR gene). The sequence data include the nucleotide residues of genomic DNA or cDNA (complementary DNA) and the amino acid residues of receptor proteins. Recently, functional data, including gene expression, molecular modelling and activity regulation, have been added as a new category of information about the receptors. In addition, ORDB is continuously being expanded to include recently identified receptors, as well as new species whose ORs have been cloned and identified [16]. Currently, ORDB contains receptors from 50 different species with complete genomic repertoires for several species, including mouse, rat and human. It represents the work of more than 100 laboratories around the world.

**Figure 1:**
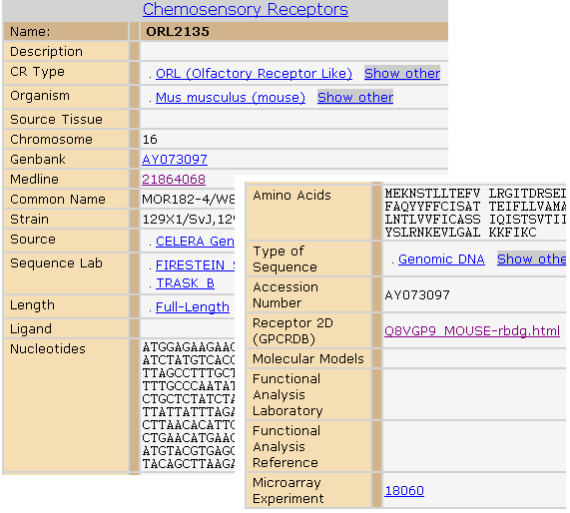
**SenseLab ORDB archiving the olfactory receptors**. This sample webpage lists the attributes (Left column) and the available values (Right column) for a receptor. The data include many descriptive attributes (such as the organism and the sequence lab), the nucleotide and amino acid sequences of the gene and its protein product, and the functional data (such as gene expression from microarray experiments). The hyperlinks lead to related pages in ORDB or other websites for detailed attribute information about the receptor. Note that the bottom part of the webpage has been cut and moved up showing the full list of the receptor attributes.

Figure 1 is an example ORDB webpage showing the information for an individual OR, ORL2135, a name based on the ORDB's nomenclature. The receptor also has a common name, e.g., MOR182-4, given by the laboratory that cloned or otherwise identified the gene. The gene for ORL2135 is located on mouse chromosome 16. Celera Genomics (Rockville, MD) is the source of the DNA sequence. The sequence type of receptors can be either genomic DNA or cDNA. In this figure, the sequence type of ORL2135 is genomic DNA. For receptors with the sequence type of cDNA, e.g., ORL466, the source tissues are usually provided. ORL466 has two putative odorant ligands: hexanol and heptanol. A URL link to ORMD (described below) is provided in the webpage. This link allows users to access the gene expression data related to the OR.

### Olfactory Receptor Microarray Database – ORMD

ORMD is a secure, Web-based repository for the OR gene expression data from the microarray experiments. The tools to access the data are the same for both regular and logged-in users. The availability of individual microarray data, however, depends on the display attribute of the experiments set by the data owners, the ownership of the data, and the logged-in status of the user. While users without login can access the data that have been made "public" by owners, users with login may access both the public data and their own private data. Only logged-in users may create projects and enter the details of data resulting from their microarray experiments. For each experiment, the gene expression datasets and the raw data files can be uploaded and stored in the database. Currently archived in the database are 31 microarray experiments from mice using the standard (Murine Genome Array U74Av2) and custom-designed Affymetrix gene-chips covering the mouse ORs. Fourteen experiments, with sample sources from the olfactory epithelium and varied body organs including brain, testes, heart, spleen, etc, have been made available to the public.

**Figure 2:**
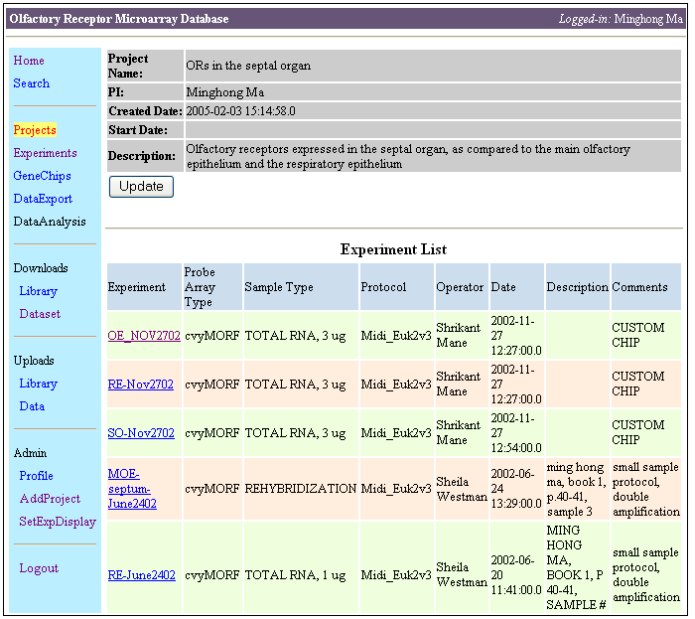
**ORMD archiving microarray experiments of the olfactory receptors**. This webpage shows a single project and the associated experiments. Listed in the left column of the page are menu items allowing users to search and browse the projects and experiments, to upload/download data files, and to export the gene expression data. The project is annotated with a PI-defined project name, PI name, archiving date, and description. The table lists all the associated experiments, with information about the array type, sample type, operator, etc. The experiment names are hyperlinks that lead to a page showing the experiment detail and gene expression data.

Figure 2 is an example webpage showing the details of an individual project. The project shown in this page investigates the OR expression patterns in the septal organ, a small island of olfactory neuroepithelium in the mouse nasal septum. The menu bar located on the left of the page allows users to use the database more effectively. The content of the page shows the project name, name of the PI, project description, and a list of associated experiments. The name of each experiment (the first column in the table) is hyperlinked to another webpage that contains details of the experiments. Each experiment is described by gene-chip (i.e., probe array type) name, sample type, protocol, and name of the operator (the technician carrying out the experiment). The owner of the data retains privileges which include modifying the content of project information and its experimental details.

**Figure 3:**
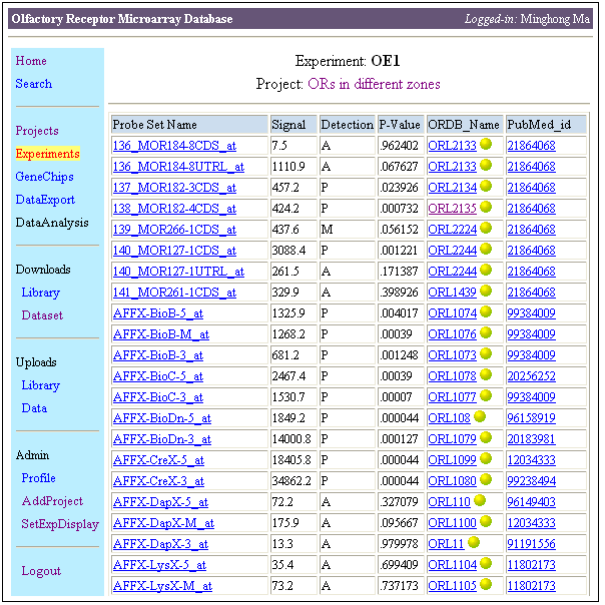
**A single experiment in ORMD archiving the gene expression data**. For each probe set, the signal intensity, detection call (A-absent; P-present), and statistical P-value are provided. For each probe set, the target gene name is provided in the column "ORDB_name" (corresponding to the name used in SenseLab). The hyperlinks on the gene name lead to the detail page of chemosensory receptors in ORDB (see Figure 1). The PubMed_IDs are hyperlinks directing users to original publications related to the genes.

Gene-chip information, including names of the probe sets on the chip and the related ORs, is stored in the database and used to annotate the expression data. Figure 3 is an example webpage showing gene expression levels in the experiment "OE1", an experiment in the project that investigates the OR expression profiles in different zones of the neuroepithelium. The table in Figure 3 shows names of the probe sets, signal intensities, and statistical calls of detections of the genes in the hybridization. It also shows the OR name used in ORDB for the probe set, with hyperlinks (implemented using receptor IDs generated by ORDB) that direct users to the webpage for that OR in ORDB. Hyperlinks showing publications related to each gene in PubMed are also provided. Users can also view the expression levels of a particular gene in all publicly accessible experiments in the database.

### Microarray experiment data upload/download

**Figure 4:**
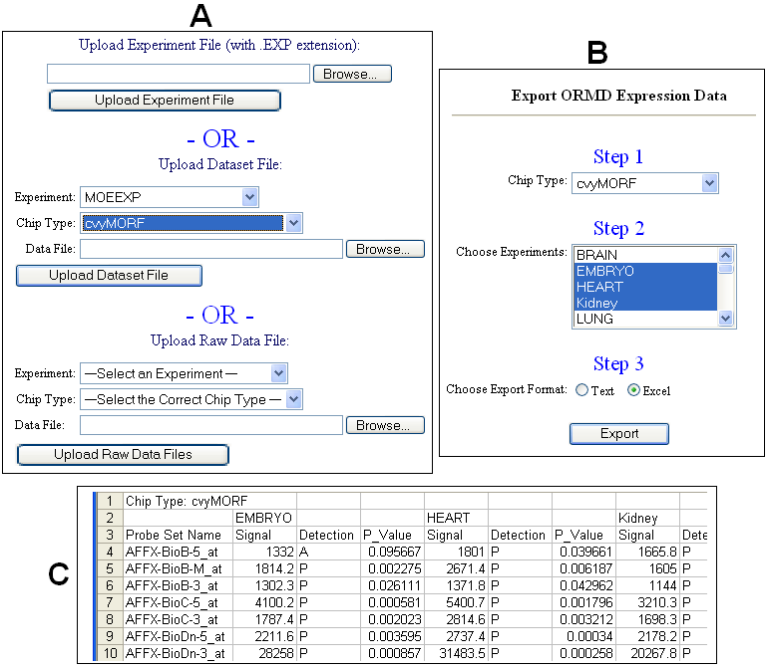
**The Web interface in ORMD for data upload and export**. A. Data upload. For each experiment, three separate uploads are carried out depending on the file type: 1) the description text file; 2) the dataset file showing the gene expressions; and 3) the raw data files. The description and dataset files are parsed by the system and the values are stored in the database. The raw data files are stored in the database as binary data type. B. Data export. The user first needs to choose a gene-chip type (Step 1), after which the dropdown list of the related experiments will be automatically refreshed. The user may select one or more experiments (Step 2) and choose an appropriate export file format, i.e., text or MS Excel (Step 3). C. A sample Excel file of gene expression data from three experiments: "Embryo," "Heart," and "Kidney".

Each microarray experiment is associated with three types of files: (1) An experiment-file that describes the details and conditions associated with hybridization; (2) A dataset file that describes the gene expression levels; and (3) Raw data files that include the hybridization image files. All these files may be uploaded into ORMD using a single Web interface (Figure 4A). The experiment and dataset files are text-based. The Web server parses these files and stores the information in the database. The probe set names in the dataset file are used to link the expression levels with individual ORs. The raw data files are stored in the Oracle database as binary data type. When an experiment file is uploaded, a corresponding "experiment" (name) will be automatically generated in the database. When the user uploads the dataset or raw data files, the corresponding experiment name is selected. The Web interface for uploading data is easy to use and intuitive.

Gene expression datasets can also be exported from ORMD. Figure 4B shows the Web interface for data export. In order to provide comparable datasets from different experiments, the data from the same gene-chip type are exported. Once the chip type is selected, the list of related experiments is automatically refreshed. Users may choose one or more datasets and export and save them (into their local computers) as text-formatted or Microsoft Excel files. Users can then use third-party tools to analyse the exported data. Figure 4C shows part of a sample Excel file of gene expression data from the experiments named "Embryo," "Heart," and "Kidney." These files describe the OR expression profiles for these three organs. In addition, the system allows users to choose multiple experiments and carry out pair-wise scatterplots to visualize the gene expression patterns between samples. Raw data files are also available for download from the experiment detail page, allowing researchers to share the data with colleagues and others in the community.

### A project and experiment management system

ORMD provides an informatics tool for researchers to manage their projects and experiments. It allows users to create/edit projects which may include multiple experiments. Individual experiments may be associated with one or more projects. This hierarchy helps researchers to systematically organize their data. It also allows efficient retrieval of the microarray datasets based on the projects.

ORMD primarily serves as a private data repository for those who will deposit data, but also provides a resource of OR gene expression information to the community. The decision as to which datasets should be made available to the public lies with the data owners (i.e., account users). While private experimental data can be made public, the publicly available data can be made private by the owners. This allows the account users the freedom to protect their unpublished data. In general, the criterion for making data public is the publication of the study in journals. The owners can also make available the unpublished data that they are willing to share with the community. The system provides the capacity to allow the public data to be adequately annotated, in particular for the MIAME-compliance of the published data [24].

## Discussion

This paper describes a database system for the storage and presentation of microarray gene expression data of olfactory receptors. The database is integrated with the olfactory databases in the SenseLab system. It is designed to facilitate experimental research in the olfactory field.

### Database integration

Investigating how odor information is transduced and processed by the olfactory system is essential to our understanding of the sense of smell. In essence, SenseLab uses the olfactory system as a model to develop informatics tools to facilitate experimental neuroscience research. The OdorDB, ORDB, and OdorMapDB in SenseLab archive the odor types, ORs, and odor map images, respectively. These olfactory databases are integrated, allowing a clear description of the chain of events from the odor stimuli to the unique activity pattern in the brain.

**Figure 5:**
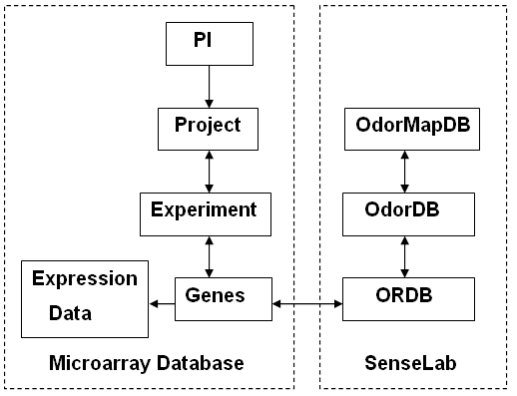
**Relationships among the major components within ORMD and among the SenseLab olfactory databases**. In ORMD, the users (PI) may create projects. The projects and experiments have a many-to-many relationship, i.e., each project may have multiple experiments and each experiment may be associated with one or more projects. Microarray gene expression data are uploaded for individual experiments and annotated with the "genes" which are probed in the particular gene-chips. The olfactory receptor "genes" in ORMD have links with the corresponding olfactory receptors archived in ORDB of the SenseLab. The receptors in ORDB are also linked to the putative odorant ligands, if there are any, in OdorDB. The odor-induced activity patterns in the brain ("odor maps") are archived in OdorMapDB.

SenseLab has been developed to be flexible. Its common architecture allows adding new databases and integrating with other systems [22]. ORMD, the creation and development of which is described in this study, is a database associated with olfaction that archives the OR gene expression data from microarray experiments. ORMD is linked to the ORDB in SenseLab through webpages providing genomic, proteomic and associated information for individual receptor genes (Figure 5). Since they store different, yet complimentary, types of data, the links that integrate ORMD and ORDB are beneficial to both databases. Users of ORMD may have easy access to the SenseLab to request gene information (for which microarray experiments have been carried out) that includes the nucleotide sequence, protein sequence, and odorant ligands known to interact (excite or inhibit) these ORs. On the other hand, users of ORDB may access microarray experiments and the related gene expression data for their OR gene of interest.

ORMD and ORDB differ not only in the type of data archived but also in the scope of user accessibility. ORDB is a knowledge-based database, with its content originating from published data. The data include the "normal" genetic information of the receptors in a given species. The volume of the ORDB grows as investigators identify new genes or extend their research into new species. On the other hand, ORMD archives gene expression data from diverse microarray experiments, dependent on tissue source and affected by biological or disease conditions. Whereas ORDB primarily provides a resource to the research community and general public (though facilities for private storage of cloned OR gene data prior to publication or deposition into GenBank exists), ORMD provides a data repository, management and sharing tool for researchers with user accounts and also allows public access to dedicated datasets.

### Informatics tools for experimental olfactory research

The eventual utility of both ORDB and ORMD in olfactory research will be evident as the microarray approach is increasingly used to investigate the gene expression patterns of ORs in the olfactory system [11,25]. Receptor genes archived in ORDB are characterized by their sequences, species, chromosome locations, etc. The expression of the receptor genes, however, depends on species, developmental stage, and tissue source. Strong expression of some genes in certain regions may help researchers uncover the relationships between animal behaviors and the stimulating odor types. Since the OR gene family contains hundreds to thousands of members in each species, the gene-chip approach provides a high-throughput, combinatorial, and powerful tool to examine the expression of the identified genes in the species simultaneously. The integrated olfactory databases described in this paper help archive and present large amounts of gene expression data, thus facilitating experimental research in understanding the molecular mechanism underlying olfactory detection and discrimination.

ORs have also been found in many other organs, such as testes, liver, and spleen. For example, the olfactory receptor hOR17-4 is found in human spermatogenic cells and may play a role in chemical communication between sperm and egg [26]. The expression of olfactory receptors in non-olfactory tissues may be due to the fact that the ORs are members of a superfamily of membrane receptors known as G-protein-coupled receptors [27]. Although the functions of ORs in other body organs remain elusive, a comprehensive investigation of ORs using microarray techniques will enhance our understanding of signal transduction in biological systems beyond olfaction.

### A generic data management system

Although ORMD is currently a data depository and management system for Affymetrix gene-chip data, it can serve as an open-source database easily adapted to house other types of microarray data. Many journals require that published microarray data conform to the MIAME Consortium. ORMD primarily serves as a private data repository, not as a portal for publication of experimental results. Although the database does not enforce the requirements set by the Consortium for private data, it allows owners to annotate the data made public according to the MIAME checklist. In general, storing good quality data always remains a high priority from system administrative as well as scientific points of view. It will be helpful that a workgroup of account users recommend and enforce the MIAME compliance as a requirement for all data made accessible to the public.

As an informatics tool, ORMD is a secure management system for microarray projects and experiments. It can be used to facilitate microarray studies in olfactory as well as other systems. The authenticated login to access the private data and the regular backup of the database ensure security of the system and protection of the data.

## Conclusion

We have described the development of ORMD and its integration with the established OR gene database ORDB. ORDB provides information on the receptor genes and proteins, while ORMD provides microarray gene expression data of the ORs. These databases include hyperlinks that connect the genes and their expression data. Together, they provide a resource for researchers using different investigative approaches to understand how mammalian organisms perceive odors.

## Availability and requirements

Project name: ORMD

Project home page: 

ORDB home page: 

Operating system(s): Platform independent

Programming language: SQL, Java

Other requirements: Access to Oracle database

License: The SQL schema is freely available from the website

Any restrictions to use by non-academics: None

## Authors' contributions

NL carried out the design and implementation of ORMD Web interface and Oracle database schema, participated in integration of ORMD and ORDB, and drafted the manuscript. CJC carried out the update and maintenance of ORDB and participated in integration of ORMD and ORDB. MM carried out the system evaluation of ORMD and was a major data contributor of the current system. All authors read and approved the final manuscript.
